# The Effects of an Acceptance and Commitment-Informed Interdisciplinary Rehabilitation Program for Chronic Airway Diseases on Health Status and Psychological Symptoms

**DOI:** 10.3389/fpsyg.2021.818659

**Published:** 2022-01-27

**Authors:** Emanuele Maria Giusti, Barbara Papazian, Chiara Manna, Valentina Giussani, Milena Perotti, Francesca Castelli, Silvia Battaglia, Pietro Galli, Agnese Rossi, Valentina Re, Karine Goulene, Gianluca Castelnuovo, Marco Stramba-Badiale

**Affiliations:** ^1^Psychology Research Laboratory, Istituto Auxologico Italiano IRCCS, Milan, Italy; ^2^Physical and Rehabilitation Medicine Unit, ASST Fatebenefratelli Sacco, Milan, Italy; ^3^Department of Psychology, Catholic University of Milan, Milan, Italy; ^4^Department of Geriatrics and Cardiovascular Medicine, IRCCS Istituto Auxologico Italiano, Milan, Italy

**Keywords:** chronic obstructive pulmonary disease (COPD), bronchiectasis, rehabilitation, acceptance and commitment based therapy, interdisciplinary program

## Abstract

**Background:**

Chronic airway diseases are prevalent and costly conditions. Interdisciplinary rehabilitation programs that include Acceptance and Commitment-based (ACT) components could be important to tackle the vicious circle linking progression of the disease, inactivity, and psychopathological symptoms.

**Methods:**

A retrospective evaluation of routinely collected data of an interdisciplinary rehabilitation program was performed. The program included group sessions including patient education, breathing exercise, occupational therapy and an ACT-based psychological treatment, and individual sessions of physical therapy. Demographic data, clinical characteristics of the patients and the values of outcome variables (health status, quality of life, anxiety, and depression) before treatment, at discharge, at 3 months, and at 6 months were extracted from medical records. Multiple imputation was employed to address missing data. Linear mixed models were employed to assess changes over time. Multivariable logistic regression was performed to assess predictors of a minimum clinically important change of health status from baseline to the 6-months follow-up.

**Results:**

Data from 31 patients with chronic obstructive pulmonary disease (COPD) and 12 patients with bronchiectasis were extracted. Health status improved from baseline to discharge to the 3 months follow-up, but not to the 6 months follow-up. Anxiety and depression improved across all the time points. Quality of life did not improve over time. Having a worse health status at baseline and fewer depressive symptoms were significantly associated with achieving a minimum clinically important change of health status at 6 months. The effects of the interdisciplinary program were not different for patients with COPD or with bronchiectasis.

**Discussion:**

Interdisciplinary programs including ACT-based components are promising treatments for the rehabilitation of patients with chronic airway diseases.

## Introduction

Chronic Obstructive Pulmonary Disease (COPD) and bronchiectasis are chronic airway diseases, mainly characterized by airflow limitation, frequent lung inflammation, presence of mucus and dyspnea (Celli et al., 2004). COPD is now the third leading cause of mortality worldwide, and the sixth leading cause of disability (Vos et al., 2020). COPD prevalence is estimated to be 13.1% worldwide, while in Europe is 12.4% (Blanco et al., 2019). Bronchiectasis has a lower prevalence, which is estimated to range from 1 in 176 to 1 in 1,492 in European countries (Chalmers et al., 2017). However, mortality at 1-year follow-up after suffering an exacerbation of bronchiectasis is between 20 and 30%, which is higher if COPD is comorbid (Finklea et al., 2010; Goeminne et al., 2014).

These conditions have a multifactorial genesis which includes genetic, environmental (e.g., exposure to pollution) and behavioral factors, in particular cigarette smoking. Although they mainly affect respiratory traits, they also cause considerable consequences on other health domains, reducing patients’ quality of life (Athanazio, 2012). Furthermore, patients who suffer from these diseases are forced to frequent hospitalizations, causing direct costs to the healthcare systems.

People suffering from airways diseases also experience psychological symptoms, such as anxiety and depression. Previous researches showed a high prevalence of psychological distress in COPD patients (Kunik et al., 2005): authors reported that 40% of COPD patients suffer from anxiety (Willgoss and Yohannes, 2013) and that female patients seem to experience greater anxiety and depression than men (Di Marco et al., 2006). Psychological distress is consequently associated with a disproportionate increase in health care utilization rates and costs: previous studies showed that COPD patients suffering from anxiety are more likely to be hospitalized and have an increased mortality risk rate (Dalal et al., 2011; Panagioti et al., 2014).

It is also known that physiological and psychological factors interact with each other, making patients’ prognosis worse (Barnes et al., 2015). These factors also interact with sociodemographic characteristics, so it becomes essential to assess their potential predictive value on the treatment’s response (De Rooij et al., 2013).

Because of their multifactorial genesis, these illnesses require a multidisciplinary approach for treatment. For this purpose, guidelines recommend an integrated approach that includes exercise training, self-management education, psychosocial interventions, breathing exercise, and occupational therapy (Gloeckl et al., 2013; GOLD, 2020; Wingårdh et al., 2020). These interventions are developed to reduce symptoms, optimize functional status, increase participation, and reduce health care costs through stabilizing or reversing systemic manifestations of the disease. These interventions have shown to significantly reduce the number of hospitalization and in improving patients’ prognosis, exercise capacity, and quality of life (Kruis et al., 2013; Poot et al., 2021). Nonetheless, these changes are often below the threshold for identifying a minimum clinically important difference, and, following a thorough review of the existing evidence, scholars have called for an improvement of these programs (Poot et al., 2021). This could be achieved if these programs are developed from an interdisciplinary rather than a multidisciplinary perspective, i.e., the different treatments are not simply juxtaposed with each other but are guided by a common framework (Giusti et al., 2017; Liang et al., 2019).

Moreover, to address the psychological issues associated with COPD and bronchiectasis, it could be useful to include an Acceptance and Commitment Therapy (ACT)-based program as a component of the interdisciplinary program. ACT-based programs are psychological interventions focused on modifying patients’ psychological flexibility, i.e., the ability to contact the present moment more fully and to change or persist in behavior when doing so serves valued ends (Hayes et al., 2006). Psychological flexibility can be evaluated assessing the six interrelated processes, or dimensions, that constitute it, namely acceptance, self as a context, cognitive defusion, contact with the present moment, values and committed action. Previous studies showed that ACT-based treatments are effective in improving both rehabilitation outcomes and patients’ quality of life scores (Graham et al., 2016).

Therefore, the primary purpose of this research was to propose and evaluate the presence of changes in clinical and psychological variables following the participation to an interdisciplinary treatment which includes an ACT-based group component for patients with COPD and bronchiectasis. A second purpose was to identify the sociodemographic, psychological, and physiological predictors of the treatment’s response.

## Materials and Methods

We performed a non-controlled study based on the analysis of routinely-collected archival data. We extracted routinely collected data from medical records of patients with COPD or bronchiectasis who were enrolled in an interdisciplinary rehabilitation program at the Rehabilitation Medicine Unit of the IRCCS Istituto Auxologico Italiano. Data were extracted from records of patients meeting the following inclusion criteria: (1) being enrolled in the interdisciplinary rehabilitation program, (2) having a medical diagnosis of COPD or bronchiectasis. We did not consider the records of patients who discontinued the program before the third session. We extracted data from medical visits, physiotherapists’ and psychologists’ records, which include information about the pre-rehabilitation demographic and clinical characteristics of the patients and the rehabilitation outcome variables. Outcome variables are collected before the start of the rehabilitation program, at the end of the program, at 3 months from the start of the rehabilitation program via phone calls, and at 6 months from the start of the rehabilitation program during routine follow-up visits. Since this study was a retrospective chart review of medical data and all patient identifiers were removed from the data before analysis, the ethical review and approval was not required. All the patients had signed a consent form regarding the use of their clinical data.

### Characteristics of the Interdisciplinary Rehabilitation Program

The interdisciplinary rehabilitation program administered at the IRCCS Istituto Auxologico Italiano was a twelve-sessions intensive program lasting 4 weeks. Physiotherapy sessions were individual, whereas the other components are administered in groups of 4–6 patients. Its components were:

• Exercise sessions. Patients underwent 12 exercise sessions of 1 h each. During the first session, patients underwent an initial functional assessment, which was performed by the referring physiotherapist by administering the COPD Assessment Test (CAT), the Borg scale, the 6-Minutes Walking Test (6MWT), and the modified Medical Research Council questionnaire (mMRC). The results of this assessment were used to adapt the content and intensity of the exercise program to the characteristics of each patient. The exercise sessions combined endurance training, resistance training and airway clearance training. Endurance training included treadmill walking, free walking, stationary cycling, stair climbing, and arm ergometer training. Exercises were performed using variable resistance and their intensity ranged from moderate (5 or 6/10 on a CR10 Borg scale) to vigorous (7 or 8/10 on a CR10 Borg scale). Resistance training was generally performed at low intensity and included weight training, leg press, and elastic band training. Airway clearance training was performed using devices such as Slow Expiration With Glottis Opened in Lateral Posture, positive expiratory pressure, temporary positive expiratory pressure, and expiratory flow accelerator. The choice of the airway clearance device was based on the patient’s clinical conditions, learning ability, and treatment compliance.
• Educational sessions. The first educational sessions is administered at the beginning of the program by a pulmonologist. Topics covered are clinical characteristics of airway diseases, their diagnosis, prognosis and treatment. A second educational session is administered by a physiatrist and covers the rationale and benefits of exercise treatment, modalities to perform exercises at home. Both sessions are co-led by a psychologist to explain psychosocial aspects related to chronic airway diseases, facilitate group discussions about the topics covered by the sessions and address potential barriers to behavior change.
• Breathing exercise. Six group sessions of breathing exercises, derived from yoga practices, are administered by licensed speech therapists. These sessions are administered to improve breath awareness and empower the patient in modifying the tendency to exert efforts to breathe, ultimately enhancing ventilatory mechanics.
• Occupational therapy. The occupational therapy component of the interdisciplinary program is administered in four sessions. These sessions are focused on teaching strategies to improve patients’ autonomy in performing basic and instrumental activities of daily life. The objectives of the treatments are tailored based on an initial evaluation of the functional limitations associated with the disease, which are assessed using the St. George Respiratory Questionnaire (see “Measurement Instrument” section). The occupational therapy program includes both educational and practical sessions aimed at providing guidance about the proper technique for the use of inhalers and adaptive equipment (e.g., sock sliders, long shoehorns) and at developing strategies to reduce shortness of breath (e.g., positioning of pillows, making pauses during basic, and instrumental activities of daily life).
• Acceptance and Commitment Therapy-based group sessions: according to the ACT principles, psychological inflexibility, in the form of the unwillingness to experience unpleasant emotions, thoughts, and bodily experiences (e.g., dyspnea), could be a core process leading to difficulties in adapting to chronic airway diseases, since it might impair motivation regarding persisting in exercise, maintain a healthy diet, and/or quit smoking. This might lead to a vicious circle linking the worsening of the disease, inactivity, and depression. The ACT-based treatment had the aim to reduce this variable. The first session was devoted to teaching defusion techniques (i.e., distancing from negative or catastrophic thoughts). The second session was devoted to discussing techniques to contact the present moment. The third session involves the exploration and identification of values. The final session is devoted to the topic of valued action and to discuss doubts and barriers to change. All the sessions were conducted using experiential exercises, metaphors and mindfulness meditation.

**FIGURE 1:**
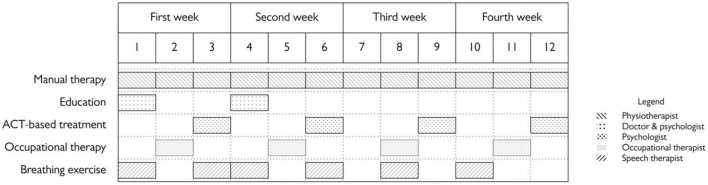
Components of the interdisciplinary program.

Patients were also provided further medical counseling at request. The organization of the program is represented in Figure 1.

### Measurement Instruments

The following information was collected from the medical records:

• Demographic (sex, age, occupation, education, and civil status) and clinical (smoke status, Body Mass Index, systolic and diastolic blood pressure, resting heart rate, GOLD classification for COPD patients, and presence of comorbidities) data.
• Spirometry results. We extracted Forced Vital Capacity (FVC), FVC in percentage predicted (FVC%), Forced Expiratory Volume in one second (FEV1), FEV1 in percentage predicted (FEV1%), the FEV/FVC% and the percentage predicted ratio FEV/FVC data (FEV/FVC% pred). Predicted values were calculated using GLI equations (Quanjer et al., 2012).
• Six-Minutes Walking Test (6MWT) data (Enright, 2003). The 6MWT is a measure of exercise tolerance for COPD patients. Patients are asked to walk as far as possible in 6 mins, with the possibility to stop or slow down when necessary. During the test physiological factors, such as oxygen saturation, blood pressure and heart frequency rate, are also recorded. The 6MWT was expressed in meters and as a percentage of the predicted normal value for age and gender, according to Enright equation (Enright and Sherrill, 1998).
• COPD Assessment Test (CAT) data. The CAT is a self-report instrument that assesses chronic airway diseases symptoms and their impact on the patient’s daily life. It is composed of eight items where patients are asked to answer on a 5-point scale. Answers are focused on symptoms such as cough, mucus, daily-life limitations, quality of sleep and energy. Scores range from 0 to 40, corresponding, respectively to the best and the worst health status. Originally developed to assess COPD symptoms, the CAT has been shown to reliably and validly measure the impact of bronchiectasis and other chronic airway diseases (Agnelo Silva de Castro et al., 2015). The Italian version of the CAT has received extensive validation which confirmed its consistency and sensitivity (Dal Negro et al., 2014). The Cronbach’s α of the CAT in our sample was 0.81.
• St. George Respiratory Questionnaire (Jones et al., 1991). The SGRQ is a self-report questionnaire investigating three respiratory-specific domains, namely symptoms (i.e., frequency and severity of respiratory symptoms such as breathlessness, coughing, and wheezing), activity (i.e., activities that cause or are limited by breathlessness) and impacts (social and psychological disturbances due to the disease). A total score can also be calculated. Total and subscale scores range from 0 to 100, with higher scores demonstrating greater impairment. The SGRQ was validated for the use with Italian patients (Carone et al., 1999).
• Modified Medical Research Council Dyspnea Scale (mMRC) (Bestall et al., 1999). The mMRC is a self-report dyspnea scale consisting of five items measuring the degree of breathlessness upon daily activities. The mMRC is scored on a range of 0 (no dyspnea or only with strenuous exertion) to 4 (dyspnea at rest).
• European Quality of life Instrument (EQ-5D) data (The EuroQol Group, 1990). The EQ-5D is a brief self-report instrument that assesses the health-related quality of life (HRQOL). It is composed of five questions, assessing five different quality of life domains: mobility, self-care, daily activities, pain, and anxiety/depression. The Italian version of the EQ-5D showed acceptable internal consistency and construct validity (Savoia et al., 2006). The EQ-5D scores were corrected using Italian norms (Scalone et al., 2013). The Cronbach’s α of the EQ-5D in our sample was 0.90.
• European Quality of life Instrument Visual Analog Scale (EQ-5D – VAS). The EQ-5D – VAS is a visual analogue scale spanning from 0 to 100, where patients have to indicate their actual perceived health status (The EuroQol Group, 1990).
• Patient Health Questionnaire (PHQ-9) data (Kroenke et al., 2001; Mazzotti et al., 2003). The PHQ-9 is a self-report instrument that assesses depressive symptoms. It is composed of nine items, based on the DSM-5 criteria to diagnose major depression. Answers were asked on a Likert scale ranging from 0 to 3, where 0 indicates “not at all” and 3 “nearly every day”. The PHQ-9 scores range from 0 to 27, with higher levels indicating higher depression severity. The scores of the Italian version of the PHQ-9 were found to be unidimensional and to have an adequate internal consistency (Shevlin et al., 2021). The Cronbach’s α of the PHQ-9 in our sample was 0.75.
• Generalized Anxiety Questionnaire (GAD-7) data (Spitzer et al., 2006). The GAD-7 is a self-report instrument that assesses anxiety symptoms. It is composed of seven items based on the DSM- IV criteria for the diagnosis of Generalized Anxiety Disorder. Answers range on three points Likert scale, where 0 indicates “not all sure” and 3 indicates “nearly every day”. GAD-7 scores range from 0 to 21, with higher values indicating higher anxiety levels. The Italian version of the GAD-7 showed adequate internal consistency and was found to be unidimensional (Shevlin et al., 2021). The Cronbach’s α of the GAD-7 in our sample was 0.82.
• Acceptance and Action Questionnaire (AAQ-II) data (Bond et al., 2011; Pennato et al., 2013). The AAQ-II is a self-report instrument aimed to assess psychological inflexibility. It is composed of seven items where patients are asked to answer on a 7-point Likert scale (from 1 = never true to 7 = always true). Higher scores indicate higher psychological inflexibility. The Italian version of the AAQ-II showed adequate internal consistency and good structural and construct validity (Pennato et al., 2013). The Cronbach’s α of the AAQ-II in our sample was 0.79.

### Statistical Analysis

Categorical variables are described using counts and percentages, continuous variables are described as medians and interquartile ranges (IQR). The internal consistency of the self-reported questionnaires was assessed by calculating their Cronbach’s α coefficient.

As a first step of our analysis, we inspected missing data mechanisms. Little’s MCAR test was performed to assess if missing data were Missing Completely At Random. Then, the associations between missing data and sex, categorical variables (education, work, and clinical diagnosis) and continuous variables were assessed using *t*-tests, ANOVAs and correlations, as appropriate. The presence of Missing At Random mechanisms were identified when *t*-tests and ANOVAs were significant or when correlations were >0.30. Since data were judged to follow a MAR mechanism, we decided to perform a multiple imputation procedure (20 datasets, 100 iterations). This approach was chosen since, contrarily to listwise deletion, it reduces bias in parameter estimates and standard errors while maintaining the original relationships among variables (van Ginkel et al., 2020). Quality of imputed data was ascertained comparing distributions of imputed datasets with the one with complete data. Multiple imputed datasets were then employed to perform the subsequent analyses.

Secondly, we checked for differences in demographic, clinical and psychological variables between patients with COPD and patients with bronchiectasis. These differences were assessed using Fisher’s exact tests, chi-square tests or Mann-Whitney tests, as appropriate.

Thirdly, we investigated the presence of changes over time in the outcome variables. Changes in psychological flexibility scores, SGRQ scores and in 6MWT results were assessed performing a separate Wilcoxon signed-rank test for each variable. Changes in general and specific quality of life, anxiety and depression were assessed using a separate linear mixed model for each variable using time (discharge vs baseline, 3 months vs baseline, and 6 months vs baseline) and diagnosis (COPD vs bronchiectasis) as fixed effects. All the models were estimated using a Restricted Maximum Likelihood estimator.

Finally, we investigated the predictors of a minimum clinically important difference at 6 months. This analysis was performed in two steps. Firstly, we performed separate bivariate logistic regressions testing the association of demographic (sex, age, and education), clinical (smoke status, BMI, presence of an exacerbation of the condition in the month before the start of the treatment, history of tumor, diabetes, history of cardiovascular diseases, and other lung diseases), spirometry (FVC and FEV1 values at the baseline), 6MWT (walked distance in meters at baseline), health status-related (baseline scores on the EQ-5D) and psychological (anxiety, depression and psychological flexibility at baseline, change in psychological flexibility from baseline to discharge) data with presence of a minimum clinically important difference at 6 months. Then, variables which were significant in bivariate analyses were included in a final multivariable logistic regression model.

The significance threshold was set at 0.05. Missing data analysis was performed using the R (version 3.6.0) package *mice* (van Buuren and Groothuis-Oudshoorn, 2011), Cronbach’s α was calculated using the R package *psych* (Revelle, 2019), linear mixed models were analyzed using the R package *lme4* (Bates et al., 2015).

## Results

We extracted data from 43 patients who participated in the interdisciplinary program. Descriptive statistics and missing data percentages are reported in Table 1. Patients with bronchiectasis were more likely to be women, to have osteoporosis, to have higher FEV1%, FEV1/FVC, and FEV1/FVC% at the spirometry performed at baseline, a higher SPO2% at the baseline 6MWT and lower mMRC scores compared to patients with COPD.

**TABLE 1:** Demographic and clinical characteristics of the sample.

		Total (*n* = 43)		COPD (*n* = 31)	Bronchiectasis (*n* = 12)	
Variable	Level	*N* (%), Median [IQR]	Missing (%)	*N* (%), Median [IQR]	*N* (%), Median [IQR]	*p*
**Demographic data**						
Female sex	Female	22 (51.2)	0 (0%)	12 (38.7)	10 (83.3)	0.02
Occupation	Unemployed	7 (16.3)	0 (0%)	4 (12.9)	3 (25.0)	
	Worker	6 (14.0)	0 (0%)	5 (16.1)	1 (8.3)	
	Retired	30 (69.8)	0 (0%)	22 (71.0)	8 (66.7)	0.56
Education	Elementary	4 (9.3)	0 (0%)	3 (9.7)	1 (8.3)	
	Middle	11 (25.6)	0 (0%)	9 (29.0)	2 (16.7)	
	High	19 (44.2)	0 (0%)	12 (38.7)	7 (58.3)	
	Degree	9 (20.9)	0 (0%)	7 (22.6)	2 (16.7)	0.7
Civil status	Unmarried	5 (11.6)	0 (0%)	3 (9.7)	2 (16.7)	
	Married	28 (65.1)	0 (0%)	21 (67.7)	7 (58.3)	
	Divorced	5 (11.6)	0 (0%)	4 (12.9)	1 (8.3)	
	Widow	5 (11.6)	0 (0%)	3 (9.7)	2 (16.7)	0.8
Age		74 [69, 77]	0 (0%)	74 [69.0, 76.5]	75.5 [69.8, 77.0]	0.48
Smoke		5 (11.6)	0 (0%)	5 (16.1)	0 (0.0)	0.34
**Clinical data and comorbidities**						
BMI		24.9 [21.2, 27.2]	0 (0%)	25 [21.2, 27.7]	23 [21.3, 24.7]	0.3
SBP		120 [120, 130]	0 (0%)	120 [120, 130]	120 [110, 130]	0.5
DBP		80 [70, 80]	0 (0%)	80 [70, 80]	75 [70, 80]	0.35
RHR		71 [64.0, 76.5]	0 (0%)	70 [62, 76]	71.5 [67.0, 77.2]	0.34
GOLD classification	A	3 (11.5)	5 (12%)			
	B	11 (42.3)				
	C	4 (15.4)				
	D	8 (30.8)				
Hypertension		18 (41.9)	0 (0%)	15 (48.4)	3 (25.0)	0.29
Tumor		15 (34.9)	0 (0%)	11 (35.5)	4 (33.3)	1
Diabetes		8 (18.6)	0 (0%)	7 (22.6)	1 (8.3)	0.52
CVD		15 (34.9)	0 (0%)	12 (38.7)	3 (25.0)	0.62
Osteoporosis		4 (9.3)	0 (0%)	0 (0.0)	4 (33.3)	0.01
Dyslipidemia		6 (14.0)	0 (0%)	4 (12.9)	2 (16.7)	1
Other lung disease		8 (18.6)	0 (0%)	8 (25.8)	0 (0.0)	0.13
**Spirometry results**						
FVC		2.3 [1.9, 2.8]	7 (16)	2.4 [1.8, 3.0]	2.2 [2.0, 2.4]	0.63
FVC%		77 [66.8, 87.4]	7 (16)	75 [64, 84]	86 [77.0, 88.2]	0.1
FEV1		1.6 [1.1, 1.9]	7 (16)	1.5 [0.9, 1.9]	1.7 [1.6, 1.9]	0.19
FEV1%		71 [53.8, 86.0]	7 (16)	59 [42, 75]	87 [78.5, 90.0]	<0.01
FEV1/FVC%		66.8 [53.7, 78.1]	8 (19)	63 [50.9, 72.3]	78.1 [75.0, 80.3]	0.01
FEV1/FVC (% pred.)		86 [70.2, 100.0]	11 (26)	77.7 [64.2, 92.0]	100.5 [99.2, 104.5]	<0.01
**Six-Minutes Walking Test results**						
Borg scale pre		3 [2, 4]	1 (2)	3 [2.0, 4.8]	2 [0.8, 3.2]	0.08
Borg scale post		3 [1.8, 4.0]	3 (7)	3 [2, 4]	2 [1.5, 4.0]	0.57
SPO2% - pre		89 [86, 92]	2 (5)	87 [85, 92]	91 [90.5, 93.2]	0.02
SPO2% - post		91 [89.0, 93.2]	3 (7)	90 [89, 93]	93 [90.0, 95.5]	0.15
Meters - pre		385 [237.0, 439.5]	1 (2)	340 [200.0, 433.5]	410 [388.8, 440.0]	0.09
Meters - post		430 [352.5, 480.0]	3 (7)	410 [300, 480]	460 [442.5, 497.5]	0.1
Predicted% - pre		77.5 [54.8, 91.5]	1 (2)	68.5 [39.8, 89.8]	86 [73.5, 97.2]	0.08
Predicted% - post		87.5 [68, 100]	3 (7)	84 [61, 99]	96 [90.5, 101.5]	0.12
**Health status and quality of life**						
mMRC Pre	0	5 (26.3)	24 (56)	5 (33.3)	0 (0.0)	0.01
	1	6 (31.6)		2 (13.3)	4 (100.0)	
	2	3 (15.8)		3 (20.0)	0 (0.0)	
	3	5 (26.3)		5 (33.3)	0 (0.0)	
CAT pre		14 [8, 17]	1 (2)	15 [8.5, 17.0]	13 [7.0, 17.5]	0.57
CAT post		8 [4, 15]	2 (5)	13 [4, 16]	6 [0.0, 8.5]	0.15
CAT 3 months		8.5 [5, 13]	7 (16)	9.5 [5.2, 13.8]	7.5 [5.5, 9.8]	0.48
CAT 6 months		12 [9, 15]	10 (23)	12.5 [9.2, 15.8]	8 [6.5, 13.5]	0.19
SGRQ symptoms pre		42 [25.2, 64.2]	5 (12)	44.5 [25.3, 65.5]	35.3 [22.6, 56.0]	0.39
SGRQ symptoms post		28.1 [17.7, 44.0]	6 (14)	28.1 [16.4, 42.0]	32.1 [21.3, 44.6]	0.4
SGRQ activities pre		59.8 [52.2, 66.2]	3 (7)	59.8 [53.5, 66.3]	59.3 [40.7, 66.2]	0.71
SGRQ activities post		53.6 [41.5, 66.2]	7 (16)	53.6 [44.7, 66.7]	53.6 [18.4, 60.2]	0.24
SGRQ impact pre		33.7 [15.9, 45.5]	3 (7)	34.1 [15.2, 45.7]	32.7 [27.4, 41.1]	0.94
SGRQ impact post		23 [9.2, 36.9]	7 (16)	24.2 [10.8, 39.5]	9.5 [9.1, 27.5]	0.18
SGRQ total pre		41 [30.3, 52.0]	3 (7)	42 [27.5, 54.5]	38.9 [32.5, 43.2]	0.67
SGRQ total post		30.6 [21.5, 45.4]	7 (16)	37.6 [21.4, 45.7]	25.4 [23.9, 27.2]	0.27
EQ-5D pre		0.8 [0.8, 0.9]	1 (2)	0.8 [0.8, 0.9]	0.8 [0.8, 0.9]	0.77
EQ-5D post		0.9 [0.8, 0.9]	7 (16)	0.8 [0.8, 0.9]	0.9 [0.8, 0.9]	0.7
EQ-5D 3 months		0.9 [0.8, 1.0]	7 (16)	0.8 [0.8, 1.0]	0.9 [0.8, 0.9]	0.51
EQ-5D 6 months		0.9 [0.8, 0.9]	9 (21)	0.8 [0.7, 0.9]	0.9 [0.8, 0.9]	0.43
EQ-5D – VAS pre		60 [50, 70]	2 (5)	60 [50, 70]	60 [55, 70]	0.73
EQ-5D – VAS post		70 [60, 75]	7 (16)	70 [60, 75]	70 [66.0, 73.8]	0.64
EQ-5D – VAS 3 months		68.5 [50, 75]	7 (16)	60 [50, 75]	70 [60, 80]	0.16
EQ-5D – VAS 6 months		65 [50, 75]	9 (21)	65 [47.5, 72.5]	65 [55, 75]	0.55
**Psychological variables**						
AAQ-II Pre		12 [10, 18]	2 (5)	12 [9.2, 15.0]	18 [11, 21]	0.38
AAQ-II Post		13 [9.2, 19.2]	9 (21)	11 [9, 17]	14 [11.5, 22.2]	0.18
PHQ-9 Pre		7 [5, 10]	2 (5)	7 [4.2, 9.0]	9 [6.0, 12.5]	0.33
PHQ-9 Post		5 [3, 7]	7 (16)	4.5 [3, 7]	7 [4.5, 9.2]	0.12
PHQ-9 3 months		5 [2, 8]	6 (14)	6 [3.5, 8.5]	5 [2, 5]	0.43
PHQ-9 6 months		3.5 [2, 6]	9 (21)	4 [2, 8]	3 [1.5, 3.5]	0.07
GAD-7 Pre		5 [3.2, 8.0]	1 (2)	5 [3, 8]	6 [5, 9]	0.25
GAD-7 Post		4.5 [3, 6]	7 (16)	4 [3, 5]	6 [5.0, 8.2]	0.04
GAD-7 3 months		3 [1, 6]	7 (16)	3 [1, 5]	6 [2, 7]	0.22
GAD-7 6 months		2 [1.2, 7.8]	9 (21)	2 [1.5, 6.5]	3 [1.5, 7.5]	0.82

*Categorical variables are described using frequencies and percentages, continuous variables using medians and Interquartile Ranges (IQR). p Values are calculated based on Fisher tests, chi square tests of Mann Whitney tests, as appropriate.*

*IQR, interquartile range; BMI, body mass index; SBP, systolic blood pressure; DBP, diastolic blood pressure; RHR, resting heart rate; CVD, cardiovascular disease; FVC, forced vital capacity; FVC%, percentage predicted FVC; FEV1, forced expiratory volume; FEV1%, percentage predicted FEV1; FEV1/FVC%, percentage predicted FEV1/FVC; CAT, COPD Assessment Test; SGRQ, St George Respiratory Questionnaire; AAQ-II, Acceptance and Action Questionnaire – II; PHQ, Patient Health Questionnaire - 9-item version; GAD, Generalized Anxiety Disorder Scale - 7-item version.*

### Missing Data Analysis

Little’s MCAR test was significant [χ^2^_(243)_ = 16,907, *p* < 0.01]. Post and follow-up scores of the mMRC and follow-up scores of the 6MWT were missing for >50% of the patients, therefore these variables were excluded from further analysis. Analysis of variables associated with missing data revealed that meters covered during the baseline 6MWT were associated with missingness at 3 months and that baseline diastolic blood pressure and meters covered during the baseline 6MWT were associated with missingness at 6 months. Based on the results of this analysis, missing data were considered to be Missing At Random. The multiple imputation procedure converged and imputed datasets were employed for the subsequent analyses.

### Analysis of Change

**FIGURE 2:**
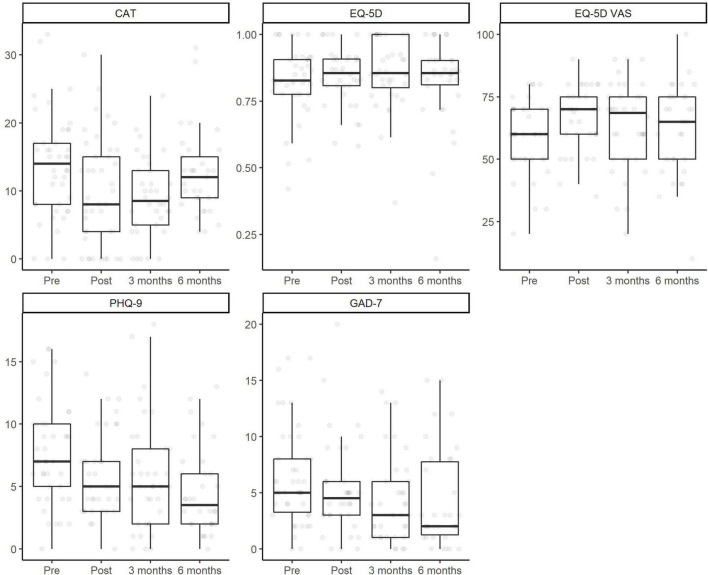
Changes over time in outcome variables.

Both the meters in the 6MWT (*U* = 60, *p* < 0.01), the SGRQ total (*U* = 628, *p* < 0.01) and subscales (Symptoms *U* = 480.5, *p* < 0.01; Activities *U* = 417.5, *p* < 0.01; Impact *U* = 584, *p* < 0.01) scores and the AAQ scores (*U* = 265.5, *p* < 0.01) improved from baseline to discharge. The results of the multilevel model are reported in Table 2. Improvement in CAT scores and in the EQ-5D – VAS was present at discharge and at 3 months, but not at 6 months. The model which analyzed changes in the EQ-5D was non-significant, showing an absence of changes over time. Conversely, both PHQ-7 and GAD-7 showed lower levels of anxiety and depression at discharge, at 3 months and at 6 months compared to baseline. The fixed effect of diagnosis (COPD vs bronchiectasis) was not significant in any model. Figure 2 displays the changes in outcome variables over time.

**TABLE 2:** Results of the mixed linear models testing the differences in the main outcomes over time.

	CAT		EQ-5D		EQ-5D VAS		PHQ-9		GAD-7	
	*B*	95% CI	*B*	95% CI	*B*	95% CI	*B*	95% CI	*B*	95% CI
Intercept	14.24	11.89–16.60*	0.82	0.76–0.87*	58.02	52.70–63.33*	7.65	6.37–8.93*	6.12	4.68–7.56*
**Time**										
Post	−3.69	−6.03 to −1.35*	0.03	−0.02 to 0.07	7.9	2.01–13.79*	−1.59	−2.99 to −0.18*	−0.84	−2.19 to 0.51
Follow-up 3 months	−4.09	−6.49 to −1.69*	0.04	−0.01 to 0.09	6.05	1.51–10.59*	−1.92	−3.37 to −0.46*	−2.15	−3.52 to −0.08*
Follow-up 6 months	−0.7	−3.19 to 1.78	−0.01	−0.06 to 0.05	1.85	−4.10 to 7.79	−2.73	−4.21 to −1.24*	−1.95	−3.34 to −0.56*
**Diagnosis**										
COPD vs Bronchiectasis	−2.48	−6.11 to 1.16	0.01	−0.06 to 0.08	4.2	−3.66 to 12.07	0.02	−1.84 to 1.87	0.91	−1.43 to 3.26

*Estimates are unstandardized coefficients.*

Estimates are unstandardized coefficients. CI, confidence interval; CAT, COPD Assessment Test; PHQ-9, Patient Health Questionnaire – 9-item version; GAD-7, Generalized Anxiety Disorder Questionnaire – 7-item version. *significant result.

### Predictors of Improvement

24 patients (*n* = 43, 55.8%), 22 (*n* = 35, 62.9%) and 17 (*n* = 32, 53.1%) patients had a minimum clinically important difference in their health status, as measured by the CAT, at discharge, at 3 months and at 6 months, respectively. Age, baseline health status, smoke status, depression levels and the difference between baseline and discharge levels of psychological inflexibility were associated with a minimum clinically important difference in CAT scores in univariate analyses. In the multivariable model, reaching a minimum clinically important difference at 6 months was predicted by baseline health status, with which had a positive association, and by baseline depression, with which had a negative association (Table 3).

**TABLE 3:** Multivariable logistic model including potential predictors of minimum clinically important difference in health status from baseline to the 6-months evaluation.

	OR	95% CI
(Intercept)	0.01	0–5.1
Age	1.01	0.01–1.18
Smoke status	0.82	0.62–1.12
CAT – baseline	1.43	1.08–1.89
PHQ – baseline	0.69	0.43–0.95
AAQ at discharge – AAQ at baseline	−0.35	0.29
*F* value		2.41
Degrees of freedom		5, 5,534.78
*P*		0.04

*OR, odds ratio; CI, confidence interval; CAT, COPD Assessment Test; PHQ, Patient Health Questionnaire; AAQ, Acceptance and Action Questionnaire.*

## Discussion

This study aimed to preliminarily assess the effects of an interdisciplinary ACT-based treatment for chronic airway diseases by analyzing changes in clinical and psychological variables in participants who attended to it, and to identify predictors of response to the treatment. The results show that patients’ health status improved over time and psychopathological symptoms decreased over time, and that baseline health status and depression were associated with achieving a minimum clinically important difference in health status at 6 months.

The potential role of ACT-based components included in interdisciplinary rehabilitation programs for patients with chronic airway diseases has been suggested before (Fernandes-James et al., 2019). ACT-based components have shown to be effective in improving the adaptation to several chronic conditions (Graham et al., 2016), and seem suitable to tackle the vicious circle linking inactivity, disease progression and psychopathological symptoms which is central for the adaptation to the disease (Hartman et al., 2013; Ramon et al., 2018). This is because ACT-based components address psychological inflexibility, which is an important factor explaining the engagement in pulmonary rehabilitation (Fernandes-James et al., 2019). Nonetheless, no study has ever addressed their efficacy. Our results, albeit of a preliminary nature, suggest that they can be an important component of interdisciplinary programs. As a note of caution, we comment that the ACT component worked in synergy with the other components and that, as a result, its specific effect is not separable from the one of the other components of the interdisciplinary program. Since interdisciplinary programs are developed by collaboration between different health professionals, the effects of their components are not entirely distinguishable.

The participation to the interdisciplinary program investigated in this study was concomitant with an improvement in patients’ health status from baseline to discharge and at 3 months, and in a reduction in psychopathological symptoms up to 6 months. Given the absence of a control group, these results should be interpreted with caution. Nevertheless, since COPD and bronchiectasis are chronic and progressive diseases, the improvement of health status in the short and medium term is of considerable clinical importance (GOLD, 2020). Similarly, improvement of anxious-depressive symptoms is crucial for optimal management of the disease (Zareifopoulos et al., 2019). Notably, the changes in health status and psychopathological symptoms did not differ between patients with COPD and patients with bronchiectasis, suggesting that both categories of patients may benefit from this treatment.

Having a worse health status at baseline was associated with more marked improvements. This suggests that interdisciplinary programs such as the one investigated in this study are particularly suitable for patients with more severe initial symptoms, as they are likely to achieve greater improvement in the long term. In contrast, higher levels of depression at the start of the program were associated with less improvement. This may be explained by the fact that depression, in general, has a negative impact on motivation for treatment and life changes needed to cope with respiratory diseases, in terms of abstinence from smoking, increased activities and being adherent to pharmacological and nonpharmacological prescriptions (Volpato et al., 2021).

The main limitation of this study is the absence of a control group. Since this study is a retrospective review of chart data, these results should be only used as the basis for further studies on the effects of ACT-based interdisciplinary treatments. In addition, the presence of missing data made it impossible to assess differences in aerobic capacity and endurance measured by the 6MWT. Missing data was substantial also for other variables, but their effect was controlled using the multiple imputation procedure.

In conclusion, this retrospective study shows that intensive interdisciplinary rehabilitation programs including an ACT-based component are promising treatments that can be employed to improve the health and psychological status of patients suffering from chronic airway diseases.

## Data Availability Statement

The raw data supporting the conclusion of this article will be made available by the authors, without undue reservation.

## Ethics Statement

Ethical review and approval was not required for the study on human participants in accordance with the local legislation and institutional requirements. The patients/participants provided their written informed consent to participate in this study.

## Author Contributions

EG conceived the study, performed the statistical analyses, and participated in the drafting of the manuscript. BP conceived the study, aided in interpreting the results, participated in the drafting of the manuscript, and conceived the study. CM participated in the drafting of the manuscript. VG, FC, SB, PG, AR, VR, KG, and GC reviewed and edited the manuscript. MP extracted the data from the medical records. MS-B was in charge of overall direction and reviewed and edited the manuscript. All authors provided critical feedback and helped to shape the research, analysis and manuscript, and approved the submitted version.

## Conflict of Interest

The authors declare that the research was conducted in the absence of any commercial or financial relationships that could be construed as a potential conflict of interest.

## Publisher’s Note

All claims expressed in this article are solely those of the authors and do not necessarily represent those of their affiliated organizations, or those of the publisher, the editors and the reviewers. Any product that may be evaluated in this article, or claim that may be made by its manufacturer, is not guaranteed or endorsed by the publisher.
