# Corrigendum: The effects of an acceptance and commitment-informed interdisciplinary rehabilitation program for chronic airway diseases on health status and psychological symptoms

**DOI:** 10.3389/fpsyg.2023.1168593

**Published:** 2023-03-07

**Authors:** Emanuele Maria Giusti, Barbara Papazian, Chiara Manna, Valentina Giussani, Milena Perotti, Francesca Castelli, Silvia Battaglia, Pietro Galli, Agnese Rossi, Valentina Re, Karine Goulene, Gianluca Castelnuovo, Marco Stramba-Badiale

**Affiliations:** ^1^Psychology Research Laboratory, Istituto Auxologico Italiano IRCCS, Milan, Italy; ^2^Physical and Rehabilitation Medicine Unit, ASST Fatebenefratelli Sacco, Milan, Italy; ^3^Department of Psychology, Catholic University of Milan, Milan, Italy; ^4^Department of Geriatrics and Cardiovascular Medicine, IRCCS Istituto Auxologico Italiano, Milan, Italy

**Keywords:** chronic obstructive pulmonary disease (COPD), bronchiectasis, rehabilitation, acceptance and commitment based therapy, interdisciplinary program

In the published article, there was an error in the affiliations of author Emanuele Maria Giusti. The second affiliation “Department of Psychology, Catholic University of Milan, Milan, Italy” was erroneously added. Emanuele Maria Giusti should just have affiliation 1.

In the published article, there was an error in affiliation 1. Instead of “Clinical Psychology Research Laboratory, Istituto Auxologico Italiano IRCCS, Milan, Italy,” it should be “Psychology Research Laboratory, Istituto Auxologico Italiano IRCCS, Milan, Italy.”

The authors apologize for this error and state that this does not change the scientific conclusions of the article in any way. The original article has been updated.

